# Rationale, Design, and Methods for the Sedentary Behavior Reduction in Pregnancy Intervention (SPRING): Protocol for a Pilot and Feasibility Randomized Controlled Trial

**DOI:** 10.2196/48228

**Published:** 2023-06-14

**Authors:** Bethany Barone Gibbs, Andrea C Kozai, Shannon N McAdoo, Meghan C Bastyr, Kelliann D Davis, Alisse Hauspurg, Janet M Catov

**Affiliations:** 1 Department of Epidemiology and Biostatistics West Virginia University School of Public Health Morgantown, WV United States; 2 Department of Health and Human Development University of Pittsburgh Pittsburgh, PA United States; 3 Department of Exercise Science University of South Carolina Columbia, SC United States; 4 Department of Obstetrics and Gynecology University of Pittsburgh Pittsburgh, PA United States; 5 Magee-Womens Research Institute Pittsburgh, PA United States

**Keywords:** pregnancy, behavioral intervention, pilot and feasibility trial, sedentary behavior, physical activity, mobile phone

## Abstract

**Background:**

Adverse pregnancy outcomes (APOs) identify cardiovascular disease risk, but few effective interventions are available. High sedentary behavior (SED) has recently been associated with APOs, but very few randomized controlled trials (RCTs) have tested SED reduction in pregnancy.

**Objective:**

The Sedentary Behavior Reduction in Pregnancy Intervention (SPRING) pilot and feasibility RCT addresses this gap by testing the feasibility, acceptability, and preliminary pregnancy health effects of an intervention to reduce SED in pregnant women. The objective of this manuscript is to describe the rationale and design of SPRING.

**Methods:**

Pregnant participants (n=53) in their first trimester, who are at risk for high SED and APO and without contraindications, are randomized in a 2:1 ratio to an intervention or control group. SED (primary outcome) and standing durations, and steps per day, are measured objectively in each trimester for 1 week with a thigh-mounted activPAL3 accelerometer. SPRING also seeks to demonstrate feasibility and acceptability while estimating preliminary effects on maternal-fetal health outcomes assessed during study visits and abstracted from medical records. The pregnancy-customized intervention promotes daily behavioral targets of less than 9 hours of SED and at least 7500 steps, achieved via increased standing and incorporating light-intensity movement breaks each hour. The multicomponent intervention provides a height-adjustable workstation, a wearable activity monitor, behavioral counseling every 2 weeks (through videoconference), and membership in a private social media group. Herein, we review the rationale, describe recruitment and screening processes, and detail the intervention, assessment protocols, and planned statistical analyses.

**Results:**

This study was funded by the American Heart Association (20TPA3549099), with a funding period of January 1, 2021, and until December 31, 2023. Institutional review board approval was obtained on February 24, 2021. Participants were randomized between October 2021 and September 2022, with final data collection planned for May 2023. Analyses and submission of results are expected for winter of 2023.

**Conclusions:**

The SPRING RCT will provide initial evidence on the feasibility and acceptability of an SED-reduction intervention to decrease SED in pregnant women. These data will inform the design of a large clinical trial testing SED reduction as a strategy to reduce APO risk.

**Trial Registration:**

ClincialTrials.gov NCT05093842; https://clinicaltrials.gov/ct2/show/NCT05093842

**International Registered Report Identifier (IRRID):**

DERR1-10.2196/48228

## Introduction

### Background

Cardiovascular disease (CVD) remains the number one health threat in women [1]. Adverse pregnancy outcomes (APOs), such as hypertensive disorders of pregnancy, gestational diabetes, and preterm birth, affect up to 20% of pregnant women and are recognized as major risk factors for latent and future CVD [2,3]. Though research to disentangle the contributions of prepregnancy risk factors from APOs on future CVD risk is ongoing, interventions that prevent APOs as a target for reducing CVD offer immediate and possibly long-term benefits. Effective preventative therapies, however, remain elusive [4]. Moderate- to vigorous-intensity physical activity reduces APO risk [5,6], but less than 1 in 4 pregnant women achieve guidelines [7]. This likely reflects that pregnant women report unique exercise barriers such as fatigue, pain, medical restriction, and concern for the baby [8].Therefore, more feasible lifestyle strategies to prevent APOs are needed.

One such strategy could be reducing sedentary behavior (SED), which is defined as low-intensity behavior that occurs in a seated, reclined, or lying posture [9]. Considering barriers to more intensive physical activity during pregnancy, replacing SED across the day with standing and light-intensity physical activities may be more achievable. Excessive SED has been related to an increased risk of CVD and mortality in general populations, even after accounting for moderate- to vigorous-intensity physical activity [10]. Though sparse data on SED during pregnancy exist, our laboratory recently completed a small cohort study among 120 pregnant women that objectively measured SED and physical activity during each trimester [11]. We found that, on average, pregnant women had approximately 9.5 hours per day of SED, which is higher than the general population [12]. Further, using trajectory analysis, women with a pattern of very high SED across pregnancy (approximately 11 hours per day) versus low SED (approximately 8 hours per day) had 6.76 (95% CI 1.20-38.14) higher odds of experiencing an APO [11]. Of interest, women in the low and medium SED groups (approximately 8 and 9 hours per day, respectively) had no excess risk of APO, indicated a possible threshold effect. Similar threshold effects were observed for time spent standing and in the number of steps per day such that only women in the very low trajectory groups had excess risk. Importantly, moderate- to vigorous-intensity activity was not associated with APO outcomes, bolstering support for a strategy of reducing SED by increasing standing and daily steps as a novel intervention target to improve pregnancy health. Yet, few randomized clinical trials (RCTs) testing SED reduction interventions are available, especially in pregnant women.

The Sedentary Behavior Reduction in Pregnancy Intervention (SPRING) pilot and feasibility study, funded by the American Heart Association (20TPA3549099), seeks to address these research gaps. SPRING is an RCT that tests the feasibility, acceptability, and preliminary effects of a novel intervention designed to reduce SED and improve all-day activity patterns among pregnant women at high risk for APOs.

### Objectives

The SPRING study’s specific aims are to (1) evaluate the effect of SPRING versus control on objectively measured SED (*primary outcome, hypothesized to decrease*), standing (*hypothesized to increase*), and steps per day (*hypothesized to increase*); (2) evaluate the feasibility and acceptability of SPRING, including recruitment, retention, outcome assessment rates, intervention fidelity, and program evaluation; and (3) estimate the preliminary effects of SPRING versus control on maternal-fetal health outcomes including composite APOs (hypertensive disorders of pregnancy, gestational diabetes, and preterm birth), maternal blood pressure and glucose, psychosocial outcomes, gestational age at birth, and infant birth anthropometrics.

## Methods

### Study Design Overview

**Figure 1:**
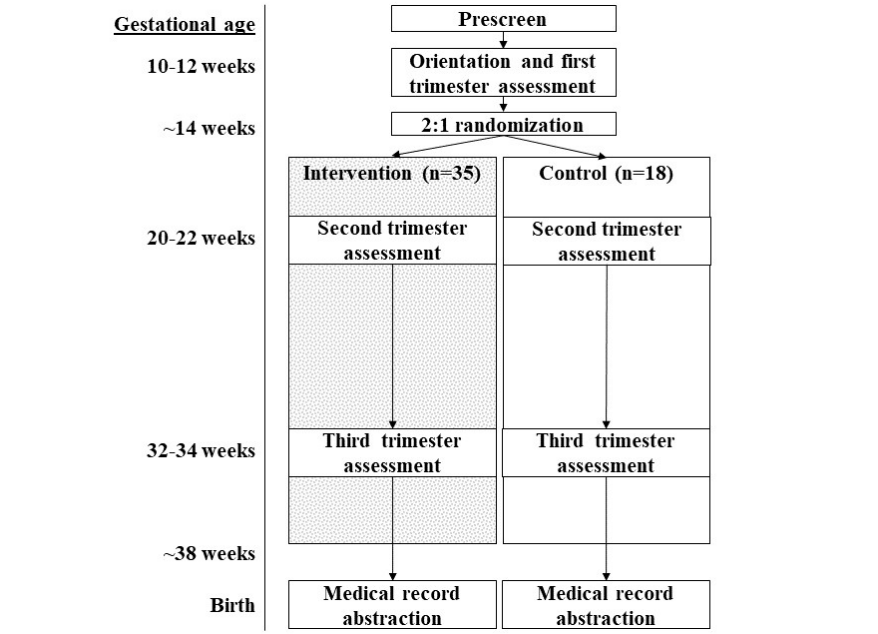
Participant flowchart across the study protocol.

In the absence of established SED-reduction interventions or guidelines in pregnant women, a pilot and feasibility RCT among pregnant women with higher risk for SED and APOs was chosen as the first logical step to determine if the intervention could successfully reduce SED by increasing standing and steps per day across pregnancy. Figure 1 describes the timeline of study assessments and intervention procedures (SPIRIT [Standard Protocol Items: Recommendations for Intervention Trials] checklist [13] available in Multimedia Appendix 1). The SPRING pilot and feasibility trial is registered on ClincialTrials.gov (NCT05093842).

Potential participants complete baseline assessments and begin the 1-week baseline activity monitoring protocol between 10 weeks 0 days (10^0^ weeks) and 12 weeks 6 days (12^6^ weeks) of gestation. Upon receipt of valid activity monitoring data with a completed time-use diary and clearance to participate from the participant’s prenatal care provider, pregnant women are randomized in a 2:1 ratio to either a multicomponent SED-reduction intervention or a usual care control group. Beginning at approximately 14 weeks of gestation, participants randomized to the intervention group begin the active intervention, which lasts through 38 weeks of gestation or until the participant gives birth, whichever occurs first. Participants randomized to the usual care control arm do not receive the intervention but are mailed a Fitbit Luxe after giving birth. Participants in both groups complete assessments in each trimester and are remunerated US $40 for completing the first trimester (baseline) assessment between 10^0^ and 12^6^ weeks, US $50 for completing the second-trimester assessment between 20^0^ and 22^6^ weeks of gestation, and US $60 for completing the third-trimester assessment between 32^0^ and 34^6^ weeks of gestation. After participants give birth, maternal-fetal outcomes are abstracted from medical records by trained clinical staff.

### Ethics Approval

All research procedures were approved by the University of Pittsburgh Human Research Protection Office (STUDY20110193).

### Eligibility Criteria

Eligibility criteria for the SPRING Study are listed in Textbox 1. We recruit participants in their first trimester of pregnancy who are at risk for high levels of SED and who have at least 1 risk factor for APO. These criteria reflect an early gestational age when we can intervene meaningfully across pregnancy and higher SED appropriate for modification (ie, reduction) to reach the levels that we have found to be associated with better pregnancy outcomes [11]. The criteria of having at least 1 risk factor for APO relate to our long-term goal to test whether our intervention can reduce APO risk; therefore, we sought to evaluate feasibility and preliminary effects in this specific population. Lastly, to allow for our exploratory outcome assessment using prenatal records of maternal-fetal outcomes, participants are required to consent to abstraction either through our affiliation with the University of Pittsburgh Medical Center or through separate medical record release if they are receiving prenatal care elsewhere.

Participants younger than 18 years or older than 45 years are excluded to avoid an inability to provide consent and more complicated pregnancies with advanced age, respectively. Those with preexisting hypertension or diabetes are excluded because existing management of these conditions could affect our study outcomes (eg, prenatal blood pressure and glucose test results). Any other contraindications to exercise during pregnancy or severe mobility limitation are also exclusionary since the intervention asks participants to increase standing and daily steps. We also require participants to receive clearance for participation in the intervention from their prenatal care provider as an additional safety precaution. Lastly, those participating in other interventional research that could confound our study outcomes are excluded to reduce bias or contamination.

**Eligibility criteria.**

**Inclusion criteria**

- Gestational age:
  Between 10 weeks 0 days and 12 weeks 6 days of gestation
- At risk for high sedentary behavior (meets at least one of the following criteria):
  Primarily sitting, full-time desk job (more than 30 hours per week)
  Primarily sitting, part-time desk job (less than 30 hours per week) and reports sitting at least half of the time during nonwork days
  Does not work and reports sitting at least three-fourths of the time
  Reports no more than 6000 steps per day from a wearable activity monitor
- Risk factor for adverse pregnancy outcome (APO; meets one of the following criteria):
  Nulliparity
  History of APO
  Prepregnancy BMI of 30 kg/m^2^ or greater
  Age of 35 years or older
- Medical record access:
  Plans to deliver at the University of Pittsburgh facility or willing to provide consent for medical record release of prenatal care and birth records

**Exclusion criteria**

- Young or advanced maternal age:
  Younger than 18 years; older than 45 years
- Chronic hypertension:
  Resting blood pressure of 140/90 mm Hg or greater or antihypertensive medication use
- Pregestational diabetes:
  Type 1 or type 2 diabetes, prior to pregnancy
- Contraindication to exercise:
  Serious medical conditions such as underlying cardiac disease, severe anemia, chronic bronchitis, poorly controlled hyperthyroidism, or seizure disorder
- Severe mobility limitation:
  Self-report of inability to walk 2 blocks or climb a flight of stairs
- No prenatal care provider clearance:
  Unable to obtain a signed permission form from a prenatal care provider to participate in the intervention
- Other research intervention:
  Participating in another health-related intervention study that could affect study outcomes
- Travel plans restricting participation:
  Plans to travel that limit ability to fully participate in the study protocol

### Recruitment and Screening

#### Recruitment Methods

Participants are recruited from within approximately 50 miles of the University of Pittsburgh’s main campus in Pittsburgh, Pennsylvania. A variety of recruitment methods are used, including the University of Pittsburgh Clinical and Translational Science Institute’s Pitt+Me research registry and targeted emails sent to the university community. In addition, recruitment messages from the study physician are sent to patients at the University of Pittsburgh Medical Center’s (UPMC) Maternal Fetal Medicine clinic who meet eligibility requirements through the MyUPMC mobile app. Finally, flyers are placed in patient-facing areas of Magee-Womens Hospital of UPMC, the largest birthing hospital in the Pittsburgh region.

#### Screening, Orientation, and Informed Consent

Interested participants are directed to complete a web-based screening questionnaire using Research Electronic Data Capture (REDCap) [14,15]. The screening questionnaire contains a detailed summary of the study, a request for electronic consent to answer screening questions, and specific questions about the candidate’s current pregnancy, risk factors for APO, SED exposure and habits, chronic diseases, medications, and contact information. Trained study staff assess initial eligibility based on the answers to the screening questionnaire and, if a candidate is deemed ineligible, they are informed at that time. Candidates who appear to be eligible during initial screening are invited to a web-based videoconference orientation where the study is described in detail, verbal informed consent is obtained, and then an electronic informed consent document is signed by the participant and study personnel conducting the informed consent. Finally, a detailed medical and reproductive history questionnaire is administered to determine final eligibility.

### Randomization

A randomization ratio of 2:1 (2 intervention to 1 control) was chosen to provide more robust data to evaluate the feasibility and acceptability of our intervention and assessment protocols [16]. To help achieve balance over time, blocks of 6 were selected, and 9 blocks of computer-generated, randomly ordered sets were placed sequentially into sealed, numbered envelopes by a blinded study personnel. These envelopes were then provided to the unblinded study personnel (principal investigator and interventionist) and kept in a secure location.

Participant randomization begins with the blinded study coordinator notifying the principal investigator that a participant is eligible and has completed all baseline assessment procedures. The study investigator then opens the next sequential envelope and records the randomization assignment and date in the randomization log. The principal investigator then contacts the participant to inform her regarding her randomization assignment, describe next steps, ensure that the participant comprehends that the intervention and assessment study procedures are separate, and reinforce the importance of continued participation in either group.

### Outcome Assessments

Study outcome assessments are conducted by trained, blinded study personnel and occur via web-based videoconference once per trimester of pregnancy (Table 1). Any necessary assessment materials are mailed to participants prior to study visits.

SED and physical activity are measured in each trimester. Objective assessment of activity patterns is conducted using a thigh-mounted activPAL3 monitor (PAL Technologies, Glasgow) worn for 24 hours over 8 days. This protocol is the best-practice methodology for field monitoring of SED and standing [17], and we have shown it to be a valid measure of moderate- to vigorous-intensity physical activity in pregnant women [18]. Participants wear the monitor on the anterior upper thigh for 8 days, with removal only for swimming activities. Concurrently, participants are asked to record periods of work, nonwork, sleep, and nonwear in a diary for accurate data processing. Participants are verbally instructed regarding procedures for correct monitor wear at each virtual assessment visit, including visual verification of correct placement. Detailed written instructions on monitor wear and diary completion are also provided. The activPAL3 24-hour event data are downloaded and cleaned by removing periods of sleep and nonwear; remaining valid wear time is used to calculate average daily durations of time spent in SED (total and in prolonged bouts of at least 30 and 60 minutes), standing, and stepping. Daily frequency of steps and sit-stand transitions are also averaged across days. Lastly, to estimate moderate- to vigorous-intensity physical activity, time with a stepping frequency of at least 75 steps per minute and 100 steps per minute are averaged across valid wear days from daily summary data. Averages are calculated overall and during working times (if applicable). Data are considered valid with at least 5 days of at least 10 hours of waking wear time recorded [19].

Self-reported SED and physical activity are assessed using the Pregnancy Physical Activity Questionnaire [20], with questions regarding proportion of time spent in SED during work and nonworking times added to improve the accuracy of self-reported SED assessment [21].

Blood pressure is assessed virtually during study assessment visits in each trimester using a validated oscillometric monitor [22]. Either a UA-611 monitor (A&D Medical) that was provided as part of prenatal care at the University of Pittsburgh Magee-Womens Hospital or a study-provided BP7250 monitor (Omron Healthcare Inc) is used. Participants use the same monitor and cuff for all visits. During the virtual assessment, participants first provide verbal confirmation of abstention from caffeine and nicotine for 1 hour prior. Then, participants place the cuff on the left upper arm and complete a supervised 5-minute quiet rest with their arm supported at chest level, back supported against a chair, and feet flat on the floor or other support with ankles and knees uncrossed. After the rest, the participant initiates the measurement by pressing the start button with the blood pressure monitor facing the video camera and visible only to the assessor (to reduce participant reactivity). The assessor leads the participant through 3 measurements, taken with a 1-minute rest in between, and records blood pressures and heart rates in the database prior to informing the participant of all values. The average of the second and third measurements is used for analysis.

Demographics, medical history, reproductive history, and medication use are collected at baseline using a standardized form. Anthropometry is assessed at baseline via self-reported height and prepregnancy weight, which are subsequently verified through medical record abstraction (described later). To capture changes that occurred following randomization, updates to demographics (eg, new employment) are reported in a questionnaire and changes to medical history or medication use (eg, a new diagnosis or starting, stopping, or changing dosage of medications) are assessed during an interval medical history review at each follow-up visit.

**Table 1:** Data collection schedule.

			Randomization						
			Baseline		Second trimester		Third trimester		Birth
**Outcomes**									
	Objective SED^a^ and activity (activPAL3)	✓		✓		✓			
	Self-reported SED and activity	✓		✓		✓			
	Blood pressure (remote observation)	✓		✓		✓			
	Demographics and medical or reproductive history	✓							
	Changes in demographics or medical history			✓		✓			
	Dietary intake	✓		✓		✓			
	Social support, SED and activity expectations, and barriers	✓		✓		✓			
	Mood, stress, depression, quality of life, and sleep	✓		✓		✓			
	Adverse events			✓		✓			
	Maternal-fetal health outcomes							✓	
**Feasibility and acceptability**									
	Recruitment, screening, and enrollment	✓							
	Expectations and experience joining SPRING^b^	✓							
	Intervention fidelity and adherence			✓		✓			
	Completion of assessment procedures	✓		✓		✓			
	Program evaluation					✓			

^a^SED: sedentary behavior.

^b^SPRING: Sedentary Behavior Reduction in Pregnancy Intervention.

Dietary intake is assessed at each visit using the Diet Screening Questionnaire, a semiquantitative questionnaire that measures typical dietary habits [23]. Psychosocial behavioral targets and outcomes are measured at baseline and each follow-up visit via web-based self-report questionnaires. Validated questionnaires assessing behavioral targets of the intervention include the Sitting during Pregnancy Barriers and Outcome Expectations Questionnaire [24], Expected Outcomes and Barriers for Habitual Physical Activity [25], the Multidimensional Scale of Perceived Social Support [26], and Social Support for Exercise [27]. Validated questionnaires assessing possible psychosocial effects of the intervention include the Profile of Mood States [28], the Centers for Epidemiologic Studies Depression Scale (CES-D) [29], the Perceived Stress Scale [30], Health-Related Quality of Life for Nausea and Vomiting during Pregnancy [31], and the Pittsburgh Sleep Quality Index [32].

Adverse events are collected systematically at follow-up assessment visits by blinded study personnel. Details of new or worsening medical conditions, including pregnancy-related adverse events, are recorded and classified regarding severity and relationship. In addition, ongoing assessment of adverse events in the intervention group is conducted by intervention staff. All adverse events are reviewed by the principal investigator and, if needed, the study physician for accuracy, possible relationship to the intervention, and to make any necessary modifications or terminate participation in the intervention.

Pregnancy health and maternal-fetal outcomes are abstracted from the participant’s medical record of prenatal visits and labor and delivery notes. Maternal outcomes extracted include gestational weight gain, clinic-measured blood pressures, pregnancy complications including APOs, glucose screening values, and tolerance tests (if available), and labor and delivery details. Fetal outcomes include gestational age at birth, anthropometrics, and complications. All abstractions are reviewed by a second reviewer and discrepancies are resolved. The study’s maternal-fetal medicine physician coinvestigator (AH) confirms all APOs and a random sample of healthy pregnancies for accuracy.

### Feasibility and Acceptability

Feasibility and acceptability are also assessed through a variety of methods across the study period (Table 2). Screening outcomes are recorded prospectively including recruitment sources and yield, eligibility rates and ineligibility reasons, and rates of enrollment. We also administer a study-developed questionnaire at baseline (prior to randomization) to assess participant expectations, motivations, and experiences when enrolling in the SPRING RCT. Rates of valid assessment completion are also prospectively recorded. Intervention fidelity with respect to delivery, receipt, and enactment [33] is assessed at each intervention contact (see Intervention Content and Schedule section). A final web-based program evaluation assesses acceptability and includes 22 short-answer, ranking, and multiple-choice questions. The evaluation is distributed after the last intervention lesson to intervention participants only and aims to evaluate participants’ perceptions regarding their health, usefulness of the intervention components, general satisfaction and acceptability of the intervention, and suggestions for improvement.

**Table 2:** Description of intervention components.

Component	Socioecological level	Description
Height-adjustable workstation [34]	Environment	A height-adjustable workstation is selected based on participant preference, occupational status, and the home or office setting. Study staff deliver and, if needed, install the workstation. This component supports the SPRING^a^ study goals to alter posture frequently and increase standing time.
Facebook group [35]	Interpersonal	A private Facebook group for SPRING participants and intervention staff offers an opportunity to discuss shared experiences of reducing SED^b^ during pregnancy and provide social support between participants. Participants are asked to join the private group after a web-based group social support lesson that includes a handful of other participants with a similar due date. Participants agree to community guidelines about respectful interaction on the page, and all activity is monitored by the intervention team. Study staff posts to the group twice per week with content aiming to provide information, engage members in discussion, and engender social support across participants.
Behavior coaching [36]	Individual or interpersonal	Participants engage in up to 13 videoconference contacts across the second and third trimesters of pregnancy. The interventionist delivers scripted lessons and check-ins (content described in more detail in Table 3) and uses motivational interviewing or problem-solving to help participants choose strategies for SED reduction and self-monitoring, set goals for standing, stepping, and movement breaks, and overcome barriers. Before each contact, the interventionist gathers the participants’ objective activity data from the previous 14 days recorded by the wearable activity monitor (described below) to guide discussions. Most lessons are delivered one-on-one, except for 1 social support lesson during the fourth contact (~18th week of gestation) that includes a small group of 2-4 intervention participants with a similar due date.
Wearable activity monitor [37]	Individual	Participants are given the choice of receiving a new Fitbit Luxe or are allowed to continue the use of a personal Fitbit or Apple Watch. This flexibility of wearable choice reflects our previous experience that participants sometimes strongly preferred to continue use of their current wearable. Participants either share credentials (Fitbit) or grant shared access to their activity data (Apple Watch) so that the interventionist can review the participants’ objective data. Participants are encouraged to wear the monitor during all waking hours and use the associated smartphone app to self-monitor their daily steps and adherence to daily movement break goals. The monitors are set up to alert participants if they have been inactive over the past hour during a participant-selected daytime interval. Participants are instructed to respond to an inactivity alert by taking a movement break, for example, a 2- to 3-minute walk or another light-intensity activity break. This component supports the study goals of breaking up prolonged SED and accumulating steps across the day.
Self-monitoring [37]	Individual	Participants are instructed to track the targeted behaviors to support behavior change and accurately report it to the interventionist during contacts. Participants are encouraged to use their wearable activity monitor app to track hourly movement breaks and daily step goals. To track standing, participants are offered paper or electronic diaries or are allowed to choose a preferred method, for example, smartphone app.

^a^SPRING: Sedentary Behavior Reduction in Pregnancy Intervention.

^b^SED: sedentary behavior.

**Table 3:** Schedule and content of the SPRING^a^ study behavioral intervention lessons.

Intervention contact	Gestational week	Intervention content
Lesson 1: Education (90 minutes)	14	Orientation to SPRING study Review of physical activity recommendations during pregnancy [36] Education about the health risks of SED^b^ with review of participant’s objective SED and steps from the baseline assessment [38] Introduction to SPRING behavioral targets Height-adjustable workstation use and ergonomic safety Use of wearable activity monitor for inactivity prompts and step tracking Choose self-monitoring strategies Goal setting for standing, movement breaks, and daily steps [39]
Check-in (15 minutes)	16	Check for contraindications Evaluation of fidelity (delivery, receipt, and enactment) [33] Discuss goal attainment and, if needed, problem-solving [40,41] Goal setting for the next 2 weeks
Lesson 2: Social support (30 minutes)	18	The only behavioral lesson that occurs as a group, including 2-4 intervention participants with a similar due date (ie, “due date group”) Discussion of social support for behavior change at the individual and community level Introduction to community-level support via the private Facebook group [35] Goal setting for community-level engagement
Check-in	20	Described in gestational week 16
Lesson 3: Know your cues (45 minutes)	22	Check-in (described above) Discuss stimulus control and environmental re-engineering [42] Evaluate daily activity patterns using the activity tracker app and apply concepts to identify inactivity patterns or creating barriers to achievement Goal setting for pregnancy-specific stimulus control strategies
Check-in	24	Described above
Lesson 4: Progress review (45 minutes)	26	Check-in (described above) Guided self-reflection on progress for standing, movement breaks, and step goals [38,43] Review of progress comparing the objective SED and steps data from second-trimester assessment to baseline levels Goal setting for improvement or maintenance of standing, movement breaks, and daily step goals in the third trimester
Check-in	24	Described above
Lesson 5: Motivation (45 minutes)	30	Check-in (described above) Introduction to concepts of internal motivation and behavior change adherence [44,45] Guided activity to identify study-specific values Goal setting and reflection on intrinsic motivation
Check-in	32	Described above
Lesson 6: Lapses do not have to be collapses (45 minutes)	34	Check-in (described above) Discussion of behavior change maintenance, relapse theory, and prevention [39] Goal setting for strategies to improve long-term maintenance of SED reduction or change
Check-in	36	Described above
Check-in	38	Described above

^a^SPRING: Sedentary Behavior Reduction in Pregnancy Intervention.

^b^SED: sedentary behavior.

### Intervention

#### Intervention Content and Schedule

SPRING developed behavioral targets informed by the specific patterns of SED, standing, and daily steps that were associated with lower APO risk in our observation cohort of pregnant women [11]. Specifically, we observed threshold effects whereby engaging in SED for approximately 9.2 hours per day or less, standing for approximately 3.8 hours per day or more, and accumulating approximately 7801 steps per day or more were the activity patterns associated with a significantly lower risk of an APO [11] along with other maternal and fetal health benefits [46-48]. We also considered additional data from our observational research regarding determinants, barriers, attitudes, and outcome expectations related to SED during pregnancy. Findings from these studies suggested that pregnancy-related physical symptoms such as nausea and fatigue along with work-related sitting were the most commonly reported barriers to reducing SED [24]. Using objectively measured SED data, we also found that desk jobs and fewer children aged 5 years or younger in the home emerged as the factors that are most strongly associated with SED during pregnancy [49]. We then used these data to adapt our existing SED-reduction interventions in nonpregnant adults [50-52] to the new population of pregnant women at high risk for SED and APOs.

**Figure 2:**
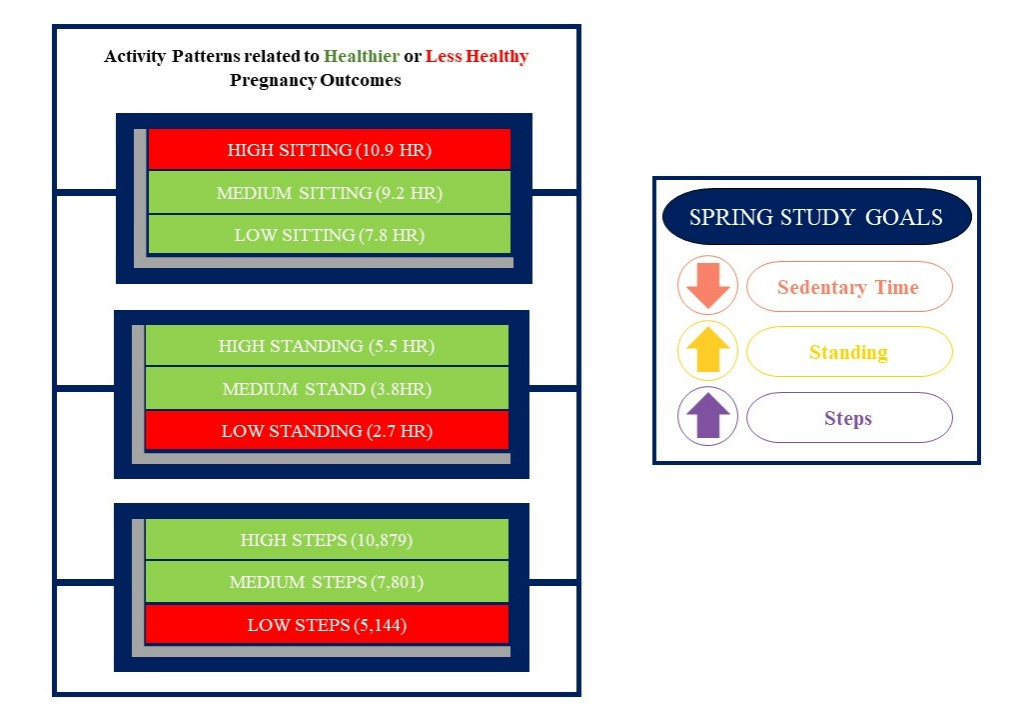
Intervention behavioral targets.

Thus, with the overall goal of reducing SED to less than 9 hours per day, we set evidence-based behavioral targets for our intervention as follows: (1) increase standing time by 2-3 hours per day with a daily total target of at least 4 hours per day; (2) increase light-intensity movement across the day, achieving at least 7500 steps per day; and (3) increase steps by at least 250 per hour (up to 8 hours per day). An example of the graphics used to convey these behavioral targets in the participant-facing intervention materials is presented in Figure 2. To facilitate behavior change toward these standing and step targets in our intervention group, we developed an evidence- and theory-based, multicomponent intervention strategy across 3 levels of the socioecological model (Table 2) [34,35,37,40].

The schedule and content of the behavioral coaching lessons are described in Table 3. Safety and fidelity of the intervention are assessed at each contact by measuring contraindications [36], delivery (provision of intervention components), receipt (self-report of intervention components working properly), and enactment (self-report of self-monitoring and goal achievement) [33].

#### Control Group

Participants assigned to the control group are asked to continue their typical behavior for the duration of their pregnancy and are provided with a general handout regarding recommended physical activity during pregnancy [36] but received no further intervention materials. After delivery, control participants are mailed a report of their objective activity levels that were previously measured during the study assessments and a Fitbit Luxe.

### Statistical Analysis

#### Analytic Strategy for Aim 1 and the Exploratory Aim

We will use Stata (StataCorp) for all analyses with an intention-to-treat philosophy. First, participant flow will be summarized using a Consolidated Standards of Reporting Trials diagram for randomized trials [53]. Second, descriptive statistics will be computed for variables by treatment group and time point. Third, the baseline measures will be compared between the 2 groups using independent samples *t* tests, Wilcoxon rank sum, chi-square, or Fisher exact tests, as appropriate. While significant differences are not expected, any found will be considered in sensitivity analyses. Next, main analyses will fit a series of linear mixed models with follow-up continuous outcomes as the dependent variable (eg, SED); intervention group (intervention or control), follow-up time (eg, second and third trimester), and group × time as fixed effects; baseline value of outcomes as fixed-effect covariates; and a participant random effect to account for multiple observations from the same participant over time. Mean contrasts will estimate the between-group difference at each follow-up time point. Similar methods will be used for the other activity variables (eg, standing and steps). Analysis of the exploratory aim will use similar methods, with logistic regression for dichotomous outcomes (eg, APOs). We will first consider the use of multiple imputation to account for missing data, but we will perform additional sensitivity or exploratory analyses to ensure robustness and extend our findings, for example, complete-data-only or last-value-carried forward strategies for missing data.

#### Analytic Strategy Aim 2

For the recruitment feasibility outcomes, we will calculate frequencies of recruitment method use and yields; rates of screening contacts, consent, eligibility, and randomization; reasons for ineligibility; and characteristics of enrolled participants. Benchmarks will be 42 (80%) for retention and 40 (95%) of the retained participants for valid outcome assessments. Intervention fidelity will be calculated based on intervention contacts with an overall average benchmark of 30 (85%) of each intervention contact competed. Quantitative or semiquantitative acceptability and program evaluation data will be summarized descriptively. Open-ended questionnaires will be analyzed using a constant comparison method to identify salient categories, themes, and patterns via open coding, iterative review and codebook generation, code linking into similar categories, and final integration into themes [54].

#### Sample Size Considerations

The primary outcome of this study is a change in SED. Based on our pilot data that suggest lower SED protects women from APOs [11], we calculated sample size requirements assuming the following: a between-group SED difference of 1 hour (SD 56 minutes) per day, a 2:1 randomization ratio to enhance recruitment and increase program evaluation and feasibility data, 90% power, and α=.05. This yielded a required sample size of 42 (28 intervention and 14 control). A final sample size of up to 53 (35 intervention and 18 control), inflated by 20%, was adopted to account for attrition.

## Results

The SPRING study was funded as an American Heart Association Transformational Project Award, which began on January 1, 2021, and ends on December 31, 2023 (20TPA3549099; Principal Investigator: BBG). Institutional review board approval was obtained on February 24, 2021. Participants were recruited over approximately 1 year, with the first subject being randomized on October 4, 2021, and the final participant being randomized on September 9, 2022. The last research assessment visit occurred on January 20, 2023. Abstraction of maternal-fetal health outcomes has a planned completion date of May 31, 2023, data analysis is planned to be completed by June 30, 2023, and submission of primary outcomes to a scientific journal is planned for September 30, 2023.

## Discussion

In summary, the SPRING pilot and feasibility RCT is testing the effects of our evidence-based SED-reduction intervention to modify SED, standing, and steps as well as determining whether our approach is feasible and acceptable among pregnant women at risk for high levels of SED and APOs. The multilevel intervention targets an activity pattern that we have found to be associated with a lower risk of APOs in observational research. To achieve these targets, our intervention approach synthesized our previous findings on determinants of SED during pregnancy with successful strategies for SED reduction intervention in nonpregnant adults. Our final exploratory aim will begin to assess the effects of our intervention on pregnancy health outcomes, including APOs. These data, including our planned in-depth feasibility, acceptability, and intervention program evaluation and clinical outcomes, will be critical for designing a fully powered RCT where we can test an optimized SED-reduction intervention for pregnant individuals. This next trial will importantly have maternal-fetal health measures (eg, APOs) as the primary outcomes so that we can understand whether our approach should be recommended as an alternative to standard moderate- to vigorous-intensity physical activity recommendations of 20-30 minutes per day. If ultimately successful, our intervention that targets reducing SED through an increase in standing and daily steps during pregnancy could offer a novel, more feasible strategy for preventing APOs and improving immediate and long-term cardiovascular health in women.

## Appendix

Multimedia Appendix 1 SPIRIT (Standard Protocol Items: Recommendations for Intervention Trials) checklist.

## Abbreviations

APO: adverse pregnancy outcome
CES-D: Center for Epidemiologic Studies Depressive Scale
CVD: cardiovascular disease
RCT: randomized controlled trial
REDCap: Research Electronic Data Capture
SED: sedentary behavior
SPIRIT: Standard Protocol Items: Recommendations for Intervention Trials
SPRING: Sedentary Behavior Reduction in Pregnancy Intervention
UPMC: University of Pittsburgh Medical Center

## Data Availability

Data generated from the SPRING study will be available upon reasonable request, with the permission of study investigators, and execution of a data use agreement.
