# Supplementary material for: Rationale, Design, and Methods for the Sedentary Behavior Reduction in Pregnancy Intervention (SPRING): Protocol for a Pilot and Feasibility Randomized Controlled Trial
Source: JMIR Res Protoc. 2023 Jun 14;12:e48228. doi: 10.2196/48228 (PMC10337422; doi:10.2196/48228)
Supplement: Multimedia Appendix 1 [file resprot_v12i1e48228_app1.docx]

Multimedia Appendix 1

SPIRIT Checklist for the Sedentary Behavior Reducing in Pregnancy Intervention (SPRING) Study (NCT05093842)

|  |  |  |  |  |
| --- | --- | --- | --- | --- |
| Timepoint | Enrollment | Allocation | Post-allocation | Close-out |
|  | Screening  *10-14 weeks gestation* | Baseline  *~13 weeks gestation* | Intervention Period  *14-38 weeks gestation* | Medical Record Abstraction  *through 6 weeks postpartum* |
| Enrollment   - Eligibility screening - Informed consent - Baseline Assessment - Allocation | X  X  X | X |  |  |
| Interventions   - SPRING Intervention group - Control group |  |  |  |  |
| Assessments   - Participant demographics, baseline behaviors, and clinical characteristics - Sedentary behavior and physical activity (primary aim) - Feasibility and acceptability (secondary aim) - Clinical outcomes e.g., blood pressure, psychosocial outcomes e.g., mood, depression (exploratory) - Adverse pregnancy outcomes, labor and delivery, labor, delivery, and birth outcomes (exploratory) | X  X  X  X |  | X X  X X  X X | X  X  X |
